# Diagnostic efficiency of the SDQ for parents to identify ADHD in the UK: a ROC analysis

**DOI:** 10.1007/s00787-015-0815-0

**Published:** 2016-01-14

**Authors:** Guillermo Perez Algorta, Alyson Lamont Dodd, Argyris Stringaris, Eric A. Youngstrom

**Affiliations:** 1 The Spectrum Centre, Lancaster University, Furness Building, C73, Bailrigg, Lancaster, LA1 4YT UK; 2 Department of Child and Adolescent Psychiatry, Institute of Psychiatry, King’s College London, London, UK; 3 Department of Psychology and Psychiatry, University of North Carolina at Chapel Hill, Chapel Hill, NC USA

**Keywords:** ADHD, Screening, Evidence-based assessment, AUC

## Abstract

Early, accurate identification of ADHD would improve outcomes while avoiding unnecessary medication exposure for non-ADHD youths, but is challenging, especially in primary care. The aim of this paper is to test the Strengths and Difficulties Questionnaire (SDQ) using a nationally representative sample to develop scoring weights for clinical use. The British Child and Adolescent Mental Health Survey (*N* = 18,232 youths 5–15 years old) included semi-structured interview DSM-IV diagnoses and parent-rated SDQ scores. Areas under the curve for SDQ subscales were good (0.81) to excellent (0.96) across sex and age groups. Hyperactivity/inattention scale scores of 10+ increased odds of ADHD by 21.3×. For discriminating ADHD from other diagnoses, accuracy was fair (<0.70) to good (0.88); Hyperactivity/inattention scale scores of 10+ increased odds of ADHD by 4.47×. The SDQ is free, easy to score, and provides clinically meaningful changes in odds of ADHD that can guide clinical decision-making in an evidence-based medicine framework.

## Introduction

Attention-deficit/hyperactivity disorder (ADHD) affects 3 % of boys and 1 % of girls in the United Kingdom [1] and an estimated 5–7 % of children globally [2–4]. It is a risk factor for interpersonal and academic problems as well as higher rates of substance misuse and antisocial behavior; among adults, it predicts higher rates of driving violations, accidents, and injuries [5] as well as occupational impairment. The economic burden of ADHD extends beyond health care to education, social services and youth justice services. The estimated annual total UK cost associated with social care and education providers’ resources for ADHD in adolescents is £670 million [6]. In the UK and globally, people with ADHD are most likely to seek services from a pediatrician or general practitioner rather than a mental health specialist. This systemic issue may contribute to delayed diagnosis [7] and commencement of treatment [8]. Early, accurate identification can improve outcomes, while avoiding unnecessary medication exposure in those youths who do not have ADHD and would not likely benefit from the same interventions [9, 10]. However, significant shortcomings in ADHD screening and diagnostic practices in primary care have been recognized [11]. The Strengths and Difficulties Questionnaire (SDQ) [12] was developed to be a brief, free-of-charge tool suitable for use in primary care and the general population. Studies have established its overall psychometric qualities [13, 14], and promising results for the identification of cases with ADHD in both community and clinical samples (see Table 1 for full list of citations and study details), with areas under the curve ranging from 0.77 to 0.97, reflecting a fair to excellent diagnostic efficiency of the SDQ. However, these have generally relied on convenience samples, often with clinical diagnoses of ADHD, which typically show a kappa of 0.49 with research diagnoses [15] (a perfect agreement would equate to a kappa of 1, and chance agreement would equate to 0). ADHD rates also change with age and with gender [16], making age and sex norms important potential moderators of test accuracy. Careful evaluation of the SDQ’s performance in a nationally representative sample, evaluating its ability to identify ADHD based on semi-structured diagnostic interviews diagnoses that integrate information about school functioning, consistent with current nosological guidelines (DSM; ICD), is required.

**Table 1:** Summary of past studies reporting area under the curve for SDQ hyperactive/inattentive subscale

Study citation	N/ADHD	Gender (% male)	Age	Source population (com, clin)	Country	AUC	95 % CI	SE
[51]	27/59^a^ 100/59^b^	55	5–12	Clin Com/clin	Yemen	0.86 0.97	0.78–0.95 0.93–1.00	–
[33]	370/173^c^	73	5–17	Clin	Germany	0.77	*P* < 0.001^g^	–
[19]	240/283^d^	65	3–17	Clin	Spain	0.86	0.82–0.89	–
[52]	47/47^e^	–	–	Clin/com	Shanghai/ China	0.77	0.71–0.83	–
[34]	110/65^f^ 98/65	–	4–16	Com/clin Clin	Germany	0.94 0.77	– –	0.02 0.03
[53]	162/11^c^ 88/11	–	4–16	Com/clin Clin	Bangladesh	0.92 0.87	–	0.03 0.05
[18]	5997/236	50	7–9	Com	Norway	0.91	0.90–0.92	–

*Com* community sample, *Clin* clinical sample, *AUC* area under the curve, *SE* standard error

^a^Conduct and Hyperactivity were collapsed in a single group using DAWBA diagnoses

^b^The discriminant power of the SDQ H/I scale was judged by comparing all community subjects with those children who had been diagnosed by the DAWBA as having a conduct and hyperactivity disorder

^c^Formal diagnosis was determined by clinical diagnosis only

^d^ADHD rating scale-IV was used as a gold standard

^e^Samples matched by age and gender

^f^Comparisons were made between community sample vs. ADHD, excluding other diagnosis from comparison group

^g^Only *p* value is provided

The overarching goal of this study is to evaluate how the SDQ for Parents could help in the clinical identification of ADHD conceptualized as a discrete category, differentiating it from other sources of externalizing behavior. To do this, we evaluated how the SDQ [12] performs for ADHD screening in a nationally representative sample of children and adolescents from the UK [1]. Although this is not the first study analyzing SDQ population data [17], it is the first direct comparison of the ability of multiple SDQ scales (Total difficulties, TD vs. Hyperactive/inattention, H/I vs. Conduct problems, CD) for discriminating ADHD cases. We expected the parent SDQ TD and the H/I subscale to outperform other SDQ subscales for detecting any ADHD disorder. Due to the gender and age differences in the rates of symptoms, we also looked at whether these changed the accuracy of the SDQ with regard to ADHD status. It would be parsimonious if the scales showed consistent accuracy [18, 19] even though the mean scores might differ. If there were differences in accuracy, a nationally representative sample provides a good basis for establishing distinct sex or age-based norms. Finally, we followed the recommendations of evidence-based medicine and facilitated clinical application of the SDQ by estimating multilevel diagnostic likelihood ratios (DLRs; [20]) for SDQ score ranges to ease clinical application of the national norms to individual cases. DLRs are defined as the probability of a positive SDQ test result given ADHD divided by the probability of positive SDQ test result given non-ADHD.

## Method

### Participants and procedures

The current study used the data from The British Child and Adolescent Mental Health Survey 1999 [21], which was designed to estimate the prevalence rates based on International Classification of Diseases-10 and DSM-IV criteria. A total of 18,232 children and adolescent (5–15 years old) were recruited from England, Wales, and Scotland (see recruitment strategy details in [1]).

Trained child and adolescent psychiatrists reviewed both the verbatim accounts and the answers to the Development and Well-Being Assessment (DAWBA; see Measurement section for further detail) [22] before assigning diagnoses. All diagnoses used in this study were unmodified DSM-IV current rather than life-time diagnostic criteria.

Parents, teachers, and eligible 11–15-year-old children were invited to complete the SDQ [12], a 25 item questionnaire divided between five scales of five items each (see details in Measurement section).

### Measures

#### Development and Well-Being Assessment (DAWBA; [22])

The DAWBA is a widely used semi-structured interview that involves child and parent interviews alongside a teacher questionnaire. The child/parent interviews and teacher questionnaires assess current and recent past psychiatric symptoms and their impact on functioning in children. The DAWBA is based on diagnostic criteria (ICD-10 and DSM-IV) and focuses on anxiety disorders, depressive disorders, ADHD and conduct disorders. A clinical diagnostic rating is informed by triangulation of these three sources.

The validity of the clinical diagnoses derived from the DAWBA have been demonstrated by concordance with case note screening in a clinical sample of children aged 11–15 years [23], and with a full clinical assessment for ADHD specifically [24].

#### Strengths and Difficulties Questionnaire (SDQ; [12]): parent version

The 25-item SDQ generates scores for five subscales confirmed through factor analysis [25]: emotional problems, conduct problems, hyperactivity-inattention, peer problems, and prosocial behaviors. A total difficulties score also sums all items. The hyperactivity-inattention scale is composed by two items about inattention, two items about hyperactivity, and one item about impulsiveness—the three key symptom domains for a DSM-IV diagnosis of attention-deficit/hyperactive disorder (ADHD) [26]. The parent SDQ demonstrated good concordance with teacher and child versions, and good test–retest reliability and internal consistency [25]. Validity was demonstrated by predictive validity and high specificity in terms of psychiatric diagnoses. Sensitivity was not as high. The present study focused on the global total difficulties score (TD), and the hyperactivity/inattention (H/I) and conduct problems (CP) subscales of the parent SDQ version as predictors of “any” DAWBA ADHD diagnosis.

### Analytic plan

Nonparametric estimates of the area under the curve (AUC) from receiver operating characteristic (ROC) analyses quantified the diagnostic efficiency of the SDQ H/I, CD and TD subscale scores. A rough guideline for evaluating AUC values is: <0.70 poor, 0.70–0.79 fair; 0.80–0.89 good; and 0.90–1.00 excellent [27], although values higher than 0.90 in mental health contexts are often the result of design flaws such as comparing clinical cases to healthy controls [28].

AUCs were calculated for the target condition of any ADHD using SDQ subscale scores, to evaluate whether the TD or subscales scores (H/I and CD) were better able to discriminate youth with any ADHD disorder from other youth in the sample. Venkatraman’s permutation test compared ROC curves [29]. Moderator analyses tested whether the diagnostic efficiency for the SDQ subscale scores changed significantly when comparing males and females, and youth age groups.

Finally, we calculated diagnostic likelihood ratios (DLRs) for optimal cut-points yielding the best balance between sensitivity and specificity from the ROC curves [30]. DLRs based on optimal cut-points provide clinically useful information for predicting the likelihood of a diagnosis. DLRs of less than 1.0 indicate that the observed score is associated with lower odds; DLRs of 1.0 mean that the score does not change the odds; DLRs between 2 and 5 are a small increase of the odds and potentially clinically meaningful; DLRs between 5.0 and 10.0 are a moderate increase, and DLRs greater than 10 are often clinically decisive [31].

All analyses were done using SPSS-Version 22.0 and pROC package in R [32].

## Results

### Demographics

Table 2 presents the participant demographics split by ADHD diagnosis. We report demographics for the ADHD combined group (*n* = 264) as well subgroups characterized by inattention (*n* = 110) and by hyperactivity (*n* = 35). Mean age and family size did not differ significantly across the groups. Half of the non-ADHD group was male, whereas this was significantly higher in each of the ADHD groups, comprising over 2/3 of the sample for each. Relative to the non-ADHD group, all three ADHD groups (combined, inattention and hyperactivity) had a significantly higher percentage of white children, single parent family background, parental unemployment and mothers with no educational qualifications. The ADHD groups had also experienced three or more life events in the past year. For clinical variables, the ADHD groups reported poorer child and parent health and family functioning, as well as higher rates of neurodevelopmental problems.

**Table 2:** Demographic and clinical information

	Non-ADHD (*n* = 18,007)	ADHD combined (*n* = 264)	ADHD inattentive (*n* = 110)	ADHD hyperactivity (*n* = 35)	Statistic *F* or chi square
Age, *M* ± *SD*	10.16 ± 3.27	10.02 ± 3.09	10.07 ± 2.81	9.32 ± 2.92	1.95
Gender, male [*n* (%)]	8987 (50)	224 (85)	83 (76)	29 (85)	170.8***
Race, white [*n* (%)]	16062 (89)	255 (97)	103 (94)	29 (85)	17.46**
Life events, 3 or more [yes (%)]	2267 (13)	75 (29)	28 (26)	11 (32)	82.09***
General health, bad [*n* (%)]	1133 (6)	46 (17)	14 (13)	4 (12)	59.93***
Neurodevelopmental problem [yes, *n* (%)]	526 (3)	46 (17)	23 (21)	3 (9)	285.15***
Mother edu qualification, none [yes (%)]	3688 (21)	90 (35)	22 (20)	8 (24)	29.80***
Parent working status, no working [yes (%)]	2991 (17)	89 (34)	27 (25)	10 (29)	61.94***
Family size, 3 or more children [yes, *n* (%)]	6324 (35)	87 (33)	48 (44)	15 (44)	5.24
Single parent family [yes, *n* (%)]	4126 (23)	104 (40)	36 (33)	9 (27)	46.01***
Family functioning score, *M* ± *SD*	1.69 ± .41^a^	1.92 ± 0.49	1.80 ± 0.48	1.90 ± 0.46	31.91***
Parent GHQ score, *M* ± *SD*	1.70 ± 2.70^a^	3.12 ± 3.32	2.96 ± 3.51	2.97 ± 3.12	33.35***

*** *p* would be significant even after stringent Bonferroni correction

^a^Non-ADHD significantly different than ADHD groups

### Diagnostic efficiency statistics

The AUCs for hyperactivity/inattention, conduct problems and total difficulties scales from the SDQ ranged from good (0.81) to excellent (0.96) in male and female subsamples and at different age ranges (Table 3a). Based on pairwise comparisons between paired AUCs between scales, H/I and TD outperformed the CP scale, except among males age 14–16 years. In the group of youngest males, H/I outperformed TD, contrary to the result observed on females of the same age group, where TD outperformed H/I. With the exception of older males, as predicted, H/I and TD outperformed the CP subscale for predicting any ADHD, and there were no major differences between H/I and TD performance, in spite of the greater number of items of the TD subscale.

**Table 3:** SDQ AUC for whole sample (a) and SDQ AUC restricted to those with a positive psychiatric diagnosis–sensitivity analyses (b)

	AUC [95 % CI]		
SDQ scales parent version	Age < 10	11–13	14–16
(a)			
Male any ADHD/non-ADHD, *n*	184/4725	96/2417	55/1762
Hyperactivity/inattention	0.92 [0.91–0.94]^a,b^	0.92 [0.91–0.94]^a^	0.91 [0.89–0.94]
Conduct problems	0.81 [0.77–0.84]^c^	0.85 [0.81–0.89]^c^	0.88 [0.83–0.92]
Total difficulties	0.90 [0.89–0.92]	0.92 [0.90–0.94]	0.91 [0.88–0.94]
Female any ADHD/non-ADHD, *n*	39/4827	21/2317	12/1769
Hyperactivity/inattention	0.92 [0.88–0.97]^a,d^	0.93 [0.87–0.99]^a^	0.96 [0.94–0.99]^a^
Conduct problems	0.82 [0.75–0.90]^c^	0.83 [0.73–0.93]^c^	0.86 [0.77–0.95]^c^
Total difficulties	0.93 [0.91–0.95]	0.92 [0.85–0.98]	0.94 [0.89–0.99]

(b)			
Male any ADHD/non-ADHD, *n*	184/330	96/209	55/184
Hyperactivity/inattention	0.76 [0.72–0.80]^a,b,¥^	0.78 [0.73–0.83]^a,b^	0.75 [0.68–0.82]^a,b^
Conduct problems	0.57 [0.52–0.62]^c^	0.63 [0.56–0.70]	0.65 [0.57–0.73]
Total difficulties	0.63 [0.58–0.68]	0.65 [0.59–0.72]	0.68 [0.60–0.75]
Female any ADHD/non-ADHD, *n*	39/233	21/170	12/198
Hyperactivity/inattention	0.78 [0.70–0.85]^a,b^	0.80 [0.70–0.90]^a^	0.88 [0.80–0.95]^a^
Conduct problems	0.61 [0.52–0.71]	0.63 [0.49–0.76]	0.66 [0.49–0.83]
Total difficulties	0.64 [0.56–0.72]	0.70 [0.57–0.83]	0.77 [0.61–0.93]

Venkatraman’s test for two paired ROC curves (*n* bootstrap replications = 2000). Superscript letters represent pairwise significantly different ROC comparisons (*p* < 0.05). Note that Venkatraman’s test compares the entire curves, so it can detect overall differences in performance even when confidence intervals for point estimates overlap

^¥^Significant (*p* < 0.05) Venkatraman’s test for two unpaired ROC curves (H/I subscale male vs. female)

^a^H/I > CD

^b^H/I > TD

^c^CP < TD

^d^H/I < TD

No significant AUC differences were found between gender and age groups (*p* > 0.05) for the H/I subscale, supporting the use of a single set of cutoff scores for the entire sample.

A score of 5+ (from a possible range of 0–10) on the H/I subscale had a DLR of 2.3, and a score of 10 yielded a DLR of 21.3, reflecting a large increase in the post-test probability of any ADHD in this national community sample (Table 4).

**Table 4:** Diagnostic likelihood ratio of the SDQ I/H subscale

	Hyperactivity/inattention scale score range			
Full sample, Any ADHD prevalence (2 %)				
	0–4		5–9	10
Diagnostic likelihood ratio	0.03		2.34	21.32
Subsample with + diagnostic, any ADHD prevalence (23 %)				
	0–4	5–7	8–9	10
Diagnostic likelihood ratio	0.06	0.77	1.80	4.47

### An outpatient proxy clinical scenario: sensitivity analyses

As the sample composition of this national study could resemble the situation described by Youngstrom et al. [28], where high AUCs are the result of comparing a majority of healthy participants with a minority of clinical cases, sensitivity analyses focused only on participants who received a positive mental health diagnosis other than any ADHD in The British Child and Adolescent Mental Health Survey 1999 (e.g., *n* = 685 with any emotional diagnosis, *n* = 608 with any anxiety diagnosis, *n* = 136 with less common psychiatric diagnosis, etc.). These evaluated the ability of the SDQ to discriminate “which diagnosis” instead of a general “sick versus well” comparison. The AUCs for H/I, CP and TD scales ranged from poor (<0.70) to good (0.88) (Table 3b). This time, when running pairwise comparisons between paired AUCs between subscales, H/I consistently outperformed CP and TD subscale. CP and TD performance were fairly similar, showing a fair or poor performance discriminating any ADHD in this subsample. As with the full sample, no significant moderating effect of age and gender was observed.

**Fig. 1:**
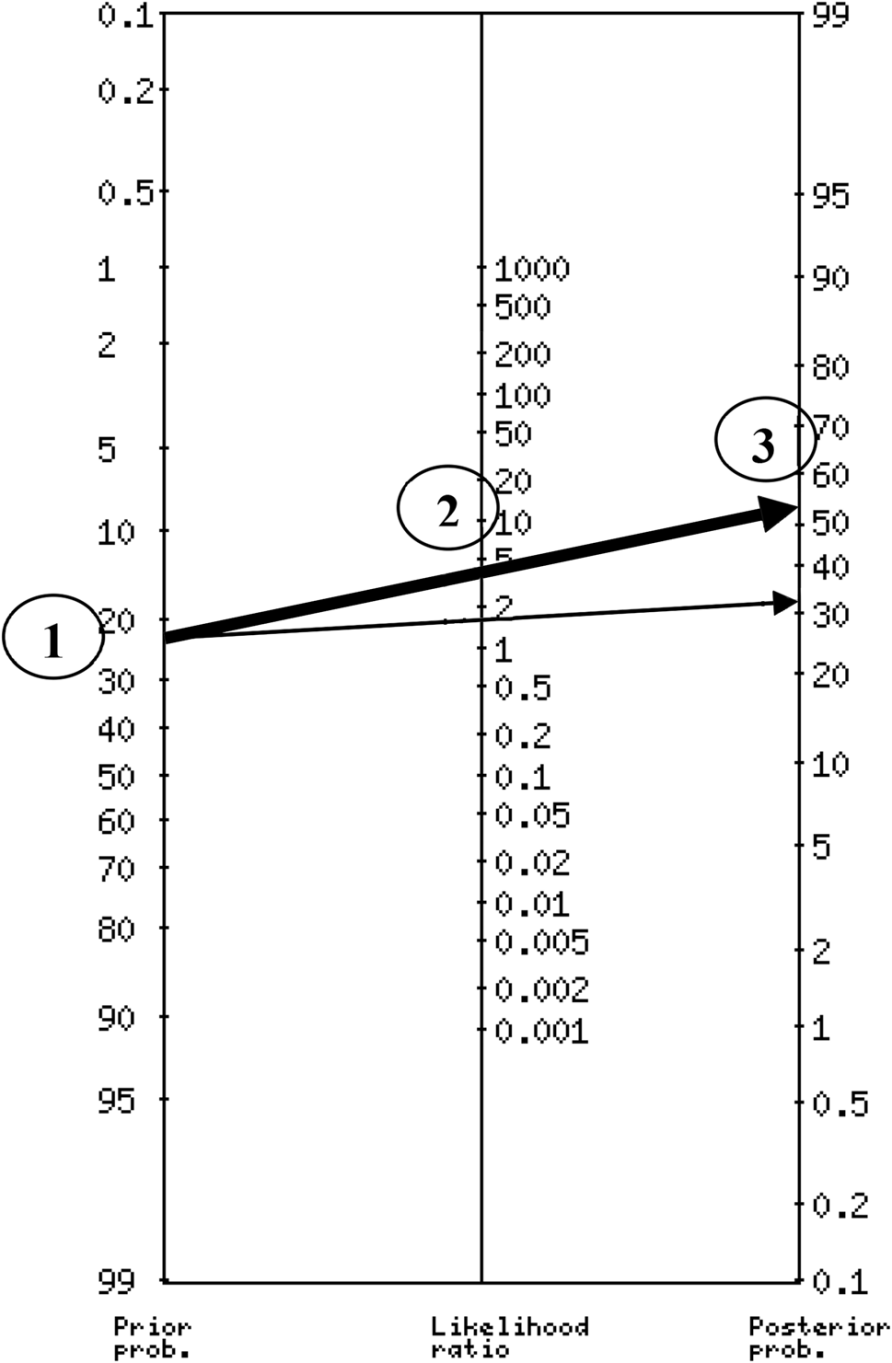
Probability Nomogram using SDQ I/H subscale. Instructions to use a nomogram: Step *1* indicates the pretest probability or estimated prevalence of a particular condition (23 % of any ADHD in this example). Step *2* in the middle axis, carries information about the associated diagnostic likelihood ratio with a particular cut score (based on Table 4, a DLR of 4.47 is associated with a score of 10). Finally, Step *3* reflects the estimate post-test probability of having any ADHD diagnosis. If a different youth obtains a different score in the SDQ I/H subscale, for example a score of 8, the only correction needed to previous steps is the identification of the appropriate DLR in Table 4. Next, trace a new line starting at the same point (identical estimated prevalence), crossing the appropriate DLR as a Step *2*, and reading the new estimated post-test probability in the last axis (see *thin arrow*)

For the comparisons limited to clinical cases, a score of 8+ (score range 0–10) in the H/I subscale produced a DLR of 1.8, and a score of 10 with a DLR of 4.47 (Table 4). Using an online calculator (http://araw.mede.uic.edu/cgi-bin/testcalc.pl) to combine the information yields a precise estimate of 57 %, and using the probability nomogram recommended in EBM provides a close, quick approximation (Fig. 1).

AUCs observed in this subsample appear similar to benchmarks from other samples that used outpatient referrals [19, 33, 34]; Table 1.

## Discussion

Results showed that the SDQ for Parents is a statistically valid tool for discriminating cases with ADHD from those without ADHD among a national representative group of youths, as well as from children experiencing other mental health diagnoses in the UK. Accuracy levels were consistent with SDQ performance discriminating psychopathology reported by Stone’s review [35], and with details provided in Table 1. Also, present results add evidence to previous findings [17], because they are based on a normative sample, addressing sampling limitations in prior work.

Results from our sensitivity analysis, which focused only on participants with a positive mental health diagnosis, confirmed the SDQ as a valid tool to detect cases with ADHD among youths meeting criteria for other disorders. Again, prior studies established a plausible range of estimates, and the present work advances clinical utility using a representative normative sample to establish weights, providing a good estimate of performance in pediatric and general practice settings.

We extended prior work by adding pairwise comparison between SDQ scales in this large community sample, testing performance of H/I, CD, and TD scales for identifying any ADHD disorder. As hypothesized, the H/I and TD scales were significantly better than the CD subscale. It is notable that the H/I scale performed similarly to the TD despite its brevity. Furthermore, in line with previous reports, no significant differences in SDQ accuracy between males and females were observed [18, 19], nor did accuracy differ between age groups [19].

Evidence-based assessment is an important component of the diagnosis and treatment of mental health problems. It can help clinicians to improve the accuracy of their diagnostic decisions and limit the influence of the bias and heuristics on clinical judgment [36]. Incorporating actuarial methods as part of the assessment process enables clinicians to integrate multiple sources of data, improving the specificity of predictions made about diagnosis and prognosis [37–39]. The SDQ discriminates cases with any ADHD disorders from those with other disorders (as observed in sensitivity analyses), showing its utility as a component of the assessment process. The current study adds to the data indicating that, in addition to identifying youth with ADHD in a representative national community sample, the SDQ can also help to identify youth with ADHD in clinical samples. This is important, as the ability to distinguish healthy youth from youth with ADHD is not as helpful as being able to distinguish youth with ADHD from youth with ODD or other externalizing symptoms. It is also one of the first studies to provide nationally representative norms and weights, combined with a semi-structured diagnostic interview to provide the criterion diagnosis. In addition to being the largest study to date, the present work also used state–of-the-art analytic methods to evaluate potential moderators of accuracy. It is also the first to present DLRs, which are crucial for clinicians to integrate the SDQ into the evidence-based assessment framework, integrating clinical findings in a way that directly guides decision-making for individual cases.

As an example, using McGee’s mnemonic [40], a likelihood ratio of 4 increases the probability of any ADHD by about 25 %. For example, with a pretest probability of 23 % (estimated prevalence of any ADHD in this subsample of youths), and a cutoff score of 10 in the H/I subscale, the post-test probability of having any ADHD would 23 + 25 = 48 %, fairly close to the more precise estimate of 57 % obtained using a probability nomogram or calculator (http://araw.mede.uic.edu/cgi-bin/testcalc.pl) to apply Bayes’ Theorem.

### Limitations

Though the SDQ has clear clinical utility, the subsamples of youth with ADHD subtypes diagnosis (particularly hyperactivity, *n* = 35) limited our ability to test SDQ performance between ADHD subtypes. Although SDQ Teacher data were gathered, teacher ratings were used as a piece of evidence in establishing the formal diagnosis using the DAWBA; thus there would be criterion contamination that would exaggerate the apparent accuracy of teacher report because it contributed to both the predictor and the criterion [41]. Future studies of the SDQ in community samples should evaluate both measures in a design that avoids criterion contamination to explore whether one out performs the other and whether there is incremental validity in combining the two [42, 43]. In this study, ADHD is conceptualized as a discrete category, an assumption that would be inconsistent with a dimensional conceptualization of ADHD. But, even when many aspects of a construct behave continuously, there are practical reasons to specify thresholds for dichotomous present/absent or treat/do not treat decisions. This is well established with both hypertension and obesity—the distribution of these is not bimodal, but thresholds are used for labeling and for treatment decisions [44].

Finally, ADHD can be conceptualized either as a source of group differences or as a constructivist variable. We note that even a constructivist definition of ADHD also has issues of reliability and measurement error. For example, patients could misread a checklist, or misconstrue the nature of the item. Clinicians frequently interpret the same responses differently—multiple studies have shown that even when presented with videotaped interviews [45] or vignettes with fixed content [46–48], clinicians apply the constructivist definitions inconsistently. Patients confront this regularly when they get a second opinion: One physician says “yes,” and the other says, “no”… so does the person have the illness or not? Kraemer [30] talked about this as resulting in imperfect reliability and validity for the diagnostic criterion, and the medical testing literature has developed methods for dealing with missing or imperfect gold standards [49, 50], recognizing that error can influence even categorical conditions with strong biological models.

The SDQ is a free, easy-to-use measure that has demonstrated utility as an ADHD disorder screening measure in community, and between youth experiencing mental health problems in the community in the UK. Current results suggest that elevated scores on the subscales of the SDQ increase the likelihood that an individual meets criteria for any ADHD, by a factor of more than 20 compared to healthy peers, and by a factor of 4.5 compared to other youths with commonly diagnosed mental health issues (as reflected by DLR in Table 4). From a clinician’s perspective, this information can be very helpful in determining whether further assessment and/or treatment is warranted as well as informing selection between treatments.

## References

[1] Ford T, Goodman R, Meltzer H (2003). The British Child and Adolescent Mental Health Survey 1999: the prevalence of DSM-IV disorders. J Am Acad Child Adolesc Psychiatry.

[2] Polanczyk G, de Lima MS, Horta BL, Biederman J, Rohde LA (2007). The worldwide prevalence of ADHD: a systematic review and metaregression analysis. Am J Psychiatry.

[3] Willcutt EG (2012). The prevalence of DSM-IV attention-deficit/hyperactivity disorder: a meta-analytic review. Neurotherapeutics J Am Soc Exp NeuroTher.

[4] Polanczyk GV, Willcutt EG, Salum GA, Kieling C, Rohde LA (2014). ADHD prevalence estimates across three decades: an updated systematic review and meta-regression analysis. Int J Epidemiol.

[5] Hechtman L, Swanson JM, Sibley M, Stehli A, Owens EB, Mitchell JT, Arnold LE, Molina BSG, Hinshaw SP, Abikoff H, Algorta GP, Howard A, Hoza B, Etcovitch J, Lakes KD, Nichols JQ, MTA Cooperative Group (under review) Functional adult outcomes 16 years after childhood diagnosis of attention-deficit/hyperactivity disorder: MTA results. JAMA Psychiatry

[6] Telford C, Green C, Logan S, Langley K, Thapar A, Ford T (2013). Estimating the costs of ongoing care for adolescents with attention-deficit hyperactivity disorder. Soc Psychiatry Psychiatr Epidemiol.

[7] Thapar AK, Thapar A (2003). Attention-deficit hyperactivity disorder. Br J Gen Pract J R Coll Gen Pract.

[8] Health NCCFM (2008) Attention deficit hyperactivity disorder: diagnosis and management of ADHD in children, young people and adults. NICE. https://www.nice.org.uk/guidance/cg72. Accessed 20 Aug 2015

[9] Hsia Y, Maclennan K (2009). Rise in psychotropic drug prescribing in children and adolescents during 1992–2001: a population-based study in the UK. Eur J Epidemiol.

[10] McCarthy S, Wilton L, Murray ML, Hodgkins P, Asherson P, Wong IC (2012). The epidemiology of pharmacologically treated attention deficit hyperactivity disorder (ADHD) in children, adolescents and adults in UK primary care. BMC Pediatr.

[11] Wolraich ML, Bard DE, Stein MT, Rushton JL, O’Connor KG (2010). Pediatricians’ attitudes and practices on ADHD before and after the development of ADHD pediatric practice guidelines. J Atten Disord.

[12] Goodman R (2001). Psychometric properties of the strengths and difficulties questionnaire. J Am Acad Child Adolesc Psychiatry.

[13] Goodman R, Renfrew D, Mullick M (2000). Predicting type of psychiatric disorder from strengths and difficulties questionnaire (SDQ) scores in child mental health clinics in London and Dhaka. Eur Child Adolesc Psychiatry.

[14] Goodman R, Ford T, Corbin T, Meltzer H (2004). Using the strengths and difficulties questionnaire (SDQ) multi-informant algorithm to screen looked-after children for psychiatric disorders. Eur Child Adolesc Psychiatry.

[15] Rettew DC, Lynch AD, Achenbach TM, Dumenci L, Ivanova MY (2009). Meta-analyses of agreement between diagnoses made from clinical evaluations and standardized diagnostic interviews. Int J Methods Psychiatr Res.

[16] Nigg JT, Barkley RA, Mash EJ, Barkley RA (2014). Attention-Deficit/Hyperactive Disorder. Child psychopathology.

[17] Rimvall MK, Elberling H, Rask CU, Helenius D, Skovgaard AM, Jeppesen P (2014). Predicting ADHD in school age when using the strengths and difficulties questionnaire in preschool age: a longitudinal general population study, CCC2000. Eur Child Adolesc Psychiatry.

[18] Ullebo AK, Posserud MB, Heiervang E, Gillberg C, Obel C (2011). Screening for the attention deficit hyperactivity disorder phenotype using the strength and difficulties questionnaire. Eur Child Adolesc Psychiatry.

[19] Carballo JJ, Rodriguez-Blanco L, Garcia-Nieto R, Baca-Garcia E (2014). Screening for the ADHD phenotype using the strengths and difficulties questionnaire in a clinical sample of newly referred children and adolescents. J Atten Disord.

[20] Jaeschke R, Guyatt GH, Sackett DL (1994). Users’ guides to the medical literature. III. How to use an article about a diagnostic test. B. What are the results and will they help me in caring for my patients? The Evidence-Based Medicine Working Group. JAMA.

[21] Meltzer H, Gatward R, Goodman R, Ford T (2000). Mental health of children and adolescents in Great Britain.

[22] Goodman R, Ford T, Richards H, Gatward R, Meltzer H (2000). The development and well-being assessment: description and initial validation of an integrated assessment of child and adolescent psychopathology. J Child Psychol Psychiatry.

[23] Goodman R, Ford T, Simmons H, Gatward R, Meltzer H (2000). Using the strengths and difficulties questionnaire (SDQ) to screen for child psychiatric disorders in a community sample. Br J Psychiatry J Ment Sci.

[24] Foreman D, Morton S, Ford T (2009). Exploring the clinical utility of the development and well-being assessment (DAWBA) in the detection of hyperkinetic disorders and associated diagnoses in clinical practice. J Child Psychol Psychiatry.

[25] Goodman A, Goodman R (2009). Strengths and difficulties questionnaire as a dimensional measure of child mental health. J Am Acad Child Adolesc Psychiatry.

[26] American Psychiatric Association (1994). Diagnostic criteria from DSM-IV.

[27] Swets JA (1988). Measuring the accuracy of diagnostic systems. Science (New York, NY).

[28] Youngstrom E, Meyers O, Youngstrom JK, Calabrese JR, Findling RL (2006). Comparing the effects of sampling designs on the diagnostic accuracy of eight promising screening algorithms for pediatric bipolar disorder. Biol Psychiatry.

[29] Venkatraman ES (2000). A permutation test to compare receiver operating characteristic curves. Biometrics.

[30] Kraemer HC (1992). Evaluating medical tests: objective and quantitative guidelines.

[31] Straus SE, Richardson WS, Glasziou P, Haynes RB (2005) Evidence-based medicine: how to practice and teach EBM

[32] Robin X, Turck N, Hainard A, Tiberti N, Lisacek F, Sanchez JC, Muller M (2011). pROC: an open-source package for R and S+ to analyze and compare ROC curves. BMC Bioinform.

[33] Becker A, Woerner W, Hasselhorn M, Banaschewski T, Rothenberger A (2004). Validation of the parent and teacher SDQ in a clinical sample. Eur Child Adolesc Psychiatry.

[34] Klasen H, Woerner W, Wolke D, Meyer R, Overmeyer S, Kaschnitz W, Rothenberger A, Goodman R (2000). Comparing the German versions of the strengths and difficulties questionnaire (SDQ-Deu) and the child behavior checklist. Eur Child Adolesc Psychiatry.

[35] Stone LL, Otten R, Engels RC, Vermulst AA, Janssens JM (2010). Psychometric properties of the parent and teacher versions of the strengths and difficulties questionnaire for 4- to 12-year-olds: a review. Clin Child Fam Psychol Rev.

[36] Garb HN, Boyle PA (2003) Understanding why some clinicians use pseudoscientific methods: findings from research on clinical judgment. In: Science and pseudoscience in clinical psychology. The Guilford Press, New York

[37] Ægisdóttir S, White MJ, Spengler PM, Maugherman AS, Anderson LA, Cook RS, Nichols CN, Lampropoulos GK, Walker BS, Cohen G (2006). The meta-analysis of clinical judgment project: fifty-six years of accumulated research on clinical versus statistical prediction. Couns Psychol.

[38] Dawes RM, Faust D, Meehl PE (1989). Clinical versus actuarial judgment. Science (New York, NY).

[39] Grove WM, Zald DH, Lebow BS, Snitz BE, Nelson C (2000). Clinical versus mechanical prediction: a meta-analysis. Psychol Assess.

[40] McGee S (2002). Simplifying likelihood ratios. J Gen Intern Med.

[41] Bossuyt PM, Reitsma JB, Bruns DE, Gatsonis CA, Glasziou PP, Irwig LM, Lijmer JG, Moher D, Rennie D, de Vet HC (2003). Towards complete and accurate reporting of studies of diagnostic accuracy: the STARD initiative. BMJ (Clinical Research ed).

[42] Youngstrom EA (2014). A primer on receiver operating characteristic analysis and diagnostic efficiency statistics for pediatric psychology: we are ready to ROC. J Pediatr Psychol.

[43] Youngstrom EA, Choukas-Bradley S, Calhoun CD, Jensen-Doss A (2015). Clinical guide to the evidence-based assessment approach to diagnosis and treatment. Cogn Behav Pract.

[44] Guyatt GH, Rennie D (2002). Users’ guides to the medical literature.

[45] Mackin P, Targum SD, Kalali A, Rom D, Young AH (2006). Culture and assessment of manic symptoms. Br J Psychiatry.

[46] Dubicka B, Carlson GA, Vail A, Harrington R (2008). Prepubertal mania: diagnostic differences between US and UK clinicians. Eur Child Adolesc Psychiatry.

[47] Jenkins MM, Youngstrom EA (2015) A randomized controlled trial of cognitive debiasing improves assessment and treatment selection for pediatric bipolar disorder. J Consul Clin Psychol 10.1037/ccp0000070 PMC4801735 26727411

[48] Jenkins MM, Youngstrom EA, Washburn JJ, Youngstrom JK (2011). Evidence-based strategies improve assessment of pediatric bipolar disorder by community practitioners. Prof Psychol Res Pract.

[49] Pepe MS (2003). The statistical evaluation of medical tests for classification and prediction.

[50] Zhou X-H, Obuchowski NA, McClish DK (2002). Statistical methods in diagnostic medicine.

[51] Alyahri A, Goodman R (2006). Validation of the arabic strengths and difficulties questionnaire and the development and well-being assessment. East Mediterr Health J = La revue de sante de la Mediterranee orientale = al-Majallah al-sihhiyah li-sharq al-mutawassit.

[52] Du Y, Kou J, Coghill D (2008). The validity, reliability and normative scores of the parent, teacher and self report versions of the strengths and difficulties questionnaire in China. Child Adolesc Psychiatry Ment Health.

[53] Mullick MS, Goodman R (2001). Questionnaire screening for mental health problems in Bangladeshi children: a preliminary study. Soc Psychiatry Psychiatr Epidemiol.

